# Comparative Analysis of Traditional and Modern Extraction Techniques for Phenolic Compounds From *Leontopodium* and Optimization of Extraction Process Using Response Surface Methodology

**DOI:** 10.1002/fsn3.72523

**Published:** 2026-10-27

**Authors:** Rule Yi, Junfeng Hao, Jian Bao, Muqier Zhao, Zhijun Wang, Zhuna Bao, Gentu Ge

**Affiliations:** ^1^ College of Grassland Science, Key Laboratory of Forage Cultivation, Processing and High Efficient Utilization of Ministry of Agriculture and Rural Affairs, Key Laboratory of Grassland Resources, Ministry of Education Inner Mongolia Agricultural University Hohhot China; ^2^ Inner Mongolia Academy of Agricultural & Animal Husbandry Sciences Hohhot Inner Mongolia People's Republic of China

**Keywords:** antioxidant activity, extraction methods, *Leontopodium*, phenolic compounds, response surface methodology, ultrasound‐assisted extraction

## Abstract

Phenolic compounds from *Leontopodium leontopodioides* (LLB) are natural bioactive constituents with potential antioxidant activity. This study compared reflux extraction (RE), microwave‐assisted extraction (MAE), and ultrasound‐assisted extraction (UAE) for recovering total flavonoids (TFC) and total phenolic content (TPC) from LLB. Based on the comparative results, UAE was further optimized using response surface methodology. The effects of habitat on TFC and TPC contents and the antioxidant activity of LLB extracts were also evaluated. Under the tested operating conditions, UAE with 70% ethanol showed the best overall extraction performance and was therefore selected for optimization. The optimized UAE conditions for TFC were an extraction time of 61 min, a solid‐to‐liquid ratio of 1:27 (g/mL), an ultrasound power of 610 W, and an extraction temperature of 58°C, yielding 40.58 ± 0.94 mg/g DW. For TPC, the optimum conditions were 40 min, 1:24 (g/mL), 430 W, and 57°C, respectively, yielding 26.38 ± 0.57 mg/g DW. LLB collected from desert grassland exhibited the highest TFC content and the strongest DPPH‐, ABTS‐, and hydroxyl‐radical‐scavenging activities, as well as the greatest reducing power. In contrast, TPC did not differ significantly among habitats (*p* > 0.05). These findings provide a basis for optimizing the extraction of phenolic constituents from LLB and support its future processing and utilization as a source of bioactive ingredients.

## Graphical abstract

Ultrasonic‐assisted extraction was identified as the most effective method for recovering TFC and TPC from LLB, with response surface analysis used to optimize the extraction conditions. The desert grassland sample had the highest TFC content (46.15 mg/g) and the strongest antioxidant activity.

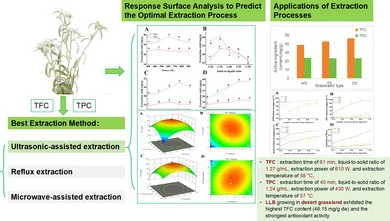

## 1 Introduction

*Leontopodium leontopodioides* (LLB) is a perennial herb in the family *Asteraceae* that is rich in bioactive constituents. Previous phytochemical studies have identified more than 50 flavonoids, including quercetin and galangin (Tauchen and Kokoska 2017; Zhang et al. 1998); more than 30 phenylpropanoids, including ethyl caffeate (Dobner et al. 2003); and more than 30 terpenoids, including betulinic acid and 15‐acetoxyisocomene (Xiao et al. 2016). Phenolic acids, such as protocatechuic acid and protocatechuic aldehyde, have also been reported in LLB (Qi et al. 2017). In addition, LLB contains polysaccharides, trace elements, and other bioactive constituents (Ablet 2005). These constituents have been associated with antibacterial, anti‐inflammatory, antidiabetic, and diuretic activities (Chen et al. 2018).

Oxidative stress, resulting from an imbalance between reactive‐species production and antioxidant defenses, can promote cellular damage and has been associated with the development of several chronic diseases (Zhan and Kurban 2010; Zhao et al. 2021a, 2021b). Phenolic constituents in LLB, particularly flavonoids and phenolic acids, may contribute to its antioxidant activity. For example, LLB extracts have been reported to exhibit scavenging activity against superoxide anion and hydroxyl radicals (Wu et al. 2013). These findings indicate that LLB may be a promising natural source of antioxidant constituents.

Conventional methods, such as reflux extraction (RE), are commonly used to recover bioactive constituents from LLB. However, these methods may require relatively long extraction times and can result in inefficient use of energy and solvents. In contrast, modern techniques, including ultrasound‐assisted extraction (UAE), microwave‐assisted extraction (MAE), and rapid solvent extraction, can enhance mass transfer and reduce extraction time. Ning et al. (2022) used UAE with ethanol to extract polyphenols from blackcurrant. Lan et al. (2023) optimized the extraction of total flavonoids from *Lycopodium clavatum* using response surface methodology (RSM). Terpinc et al. (2023) compared MAE and accelerated solvent extraction for phenolic recovery from wild strawberry leaves. Khan et al. (2025) compared conventional and modern extraction techniques and optimized UAE conditions for polyphenol recovery using RSM.

RSM is a statistical approach for optimizing processes involving multiple interacting variables. It uses regression models to describe the relationships between experimental factors and response variables, thereby facilitating the prediction and validation of optimal conditions with relatively few experimental runs (Khan et al. 2025; Yang et al. 2026). Chen et al. (2024) characterized the chemical composition of LLB water extracts and essential oil using UPLC‐Q‐Orbitrap MS and GC–MS, identifying 39 constituents, including flavonoids, phenolic acids, pentacyclic triterpenoids, oligosaccharides, and glycosides. In our previous studies, we used rapid solvent extraction to recover bioactive constituents from *Leontopodium leontopodioides* and optimized the extraction procedure (Yi et al. 2024). We also investigated the spatial and temporal distribution of its major compounds (Yi, Ge, et al. 2026; Yi, Hao, et al. 2026). Building on this chemical characterization, the present study compares reflux, microwave‐assisted, and ultrasound‐assisted extraction in terms of their recovery of total flavonoids and total phenolics. We further optimize ultrasound‐assisted extraction using a four‐factor Box–Behnken design and examine habitat‐associated variation in phenolic content and antioxidant activity. Thus, this study extends our previous work by providing method‐specific evidence on extraction performance and optimization, together with an assessment of habitat‐related variation in chemical content and antioxidant activity.

Accordingly, the aerial parts of wild LLB were used to screen suitable extraction methods. The optimized UAE protocol was subsequently applied to LLB samples collected from different habitats, and the antioxidant activities of the resulting extracts were evaluated. The study aimed to provide a basis for improving LLB extraction processes and supporting the utilization of its bioactive constituents. Considerations relevant to future scale‐up, including solvent suitability, energy consumption, and product stability, were also considered during process development. Nevertheless, translation to a larger scale will require further validation of ultrasound energy distribution, temperature control, mixing efficiency, solvent recovery, batch‐to‐batch consistency, and product integrity (Chemat et al. 2017). The findings may support the future development of standardized LLB extracts for functional‐food, nutraceutical, or functional‐feed applications.

## 2 Methods

### 2.1 Plant Materials

The plant material was identified as *Leontopodium leontopodioides* (Willd.) Beauv. (LLB). The species used in this study has not been formally assessed by or listed on the International Union for Conservation of Nature (IUCN) Red List of Threatened Species. Collection of L. leontopodioides was authorized by the relevant local authorities and conducted in accordance with applicable national and international guidelines. A voucher specimen was deposited in the Herbarium of Inner Mongolia Agricultural University (publicly accessible; voucher no. HHR2025080201) and identified by Professor Naris. Aerial parts were used exclusively as the plant material in this study.

Samples from the meadow steppe were collected in July 2024 from New Barag Left Banner, Hulunbuir City, Inner Mongolia, China (48.21°N, 118.26°E; approximately 1573 m above sea level). The site has a mean annual temperature of 0.2°C and a mid‐temperate continental monsoon climate. Its climate is characterized by long and severely cold winters, dry and windy springs, mild summers with concentrated precipitation, rapid temperature declines in autumn, and a relatively short frost‐free period. Samples from the typical steppe were collected in August 2024 from Ruizhi Town, Keshiketeng Banner, Chifeng City, Inner Mongolia, China (43.38°N, 117.26°E; 1353 m above sea level). The site has a mean annual temperature of 2°C–4°C and a temperate semi‐arid continental monsoon climate, characterized by long and cold winters, dry and windy springs, and a frost‐free period of approximately 110 days (used as the experimental material for the extraction process study). Samples from the desert steppe were collected in August 2024 from Chahar Right Middle Banner, Ulanqab City, Inner Mongolia, China (41.27°N, 112.63°E; approximately 1640 m above sea level). The site has a mean annual temperature of approximately 1.3°C and a temperate continental climate. The climate is characterized by long and cold winters, short and cool summers, marked diurnal temperature variation, and a frost‐free period of approximately 90–110 days. After collection, the plant samples were washed, shade‐dried, ground using a grinder, passed through a 60‐mesh sieve, and sealed until further analysis.

### 2.2 Reagents

Rutin, gallic acid, Folin–Ciocalteu reagent, DPPH, and ABTS were purchased from Shanghai Yuanye Technology Co. Ltd. Anhydrous ethanol, ferric chloride, hydrogen peroxide, salicylic acid, sodium nitrite, aluminum nitrate, sodium hydroxide, sodium carbonate, phosphate, potassium chloride, trichloroacetic acid, ascorbic acid, and other reagents were obtained from Sinopharm Chemical Reagent Co. Ltd. All reagents were of analytical grade. Extraction solutions were prepared using deionized water.

### 2.3 Methods for the Extraction of Polyphenols From LLB

#### 2.3.1 Reflux Extraction (RE)

LLB powder (3.0 g) was accurately weighed into a beaker and extracted with 70% ethanol at a solid‐to‐liquid ratio of 1:25 (g/mL). Reflux extraction was conducted for 15 min at 45°C, 60°C, or 75°C. The extract was filtered through filter paper, and the filtrate was brought to a final volume of 50 mL with 70% ethanol. Total flavonoid content (TFC) and total phenolic content (TPC) were determined using the respective calibration curves. Each treatment was performed in triplicate.

#### 2.3.2 Microwave‐Assisted Extraction (MAE)

LLB powder (3.0 g) was accurately weighed into a three‐necked flask containing one or two magnetic stirring bars. Extraction was performed with 70% ethanol at a solid‐to‐liquid ratio of 1:25 (g/mL). The samples were treated at 30°C for 15 min under microwave powers of 400, 500, or 600 W. After extraction, the mixture was filtered through filter paper, and the filtrate was adjusted to 50 mL with 70% ethanol. TFC and TPC were determined using the respective calibration curves. Each treatment was performed in triplicate.

#### 2.3.3 Ultrasound‐Assisted Extraction (UAE)

LLB powder (3.0 g) was accurately weighed into a beaker and extracted with 70% ethanol at a solid‐to‐liquid ratio of 1:25 (g/mL). Ultrasound‐assisted extraction was conducted at 30°C for 15 min under ultrasound powers of 450, 600, or 750 W. The extract was filtered through filter paper, and the filtrate was adjusted to 50 mL with 70% ethanol. TFC and TPC were determined using the respective calibration curves. Each treatment was performed in triplicate.

### 2.4 Determination of Phenolic Compounds

#### 2.4.1 Determination of Total Flavonoid Content

The determination of TFC content was carried out with reference to the method described by Yi et al. (2008), with minor modifications. Rutin was utilized as the reference standard, and the determination was performed using the sodium nitrite–aluminum nitrate colorimetric method. The procedure is outlined as follows: The rutin standard solutions of varying concentrations are to be measured accurately. Thereafter, 40 μL of a 5% NaNO_2_ solution is to be added to each solution, and the mixture is to be thoroughly agitated. The reaction is to be allowed to proceed for a period of 6 min. Thereafter, 40 μL of a 10% Al (NO_3_)_3_ solution is to be added, and the mixture is to be thoroughly agitated. The reaction is to be allowed to proceed for a period of 6 min. Subsequently, 400 μL of a 1 M NaOH solution and 400 μL of distilled water are to be added, and the mixture is to be thoroughly agitated. The reaction is to be allowed to stand for a period of 15 min. The measurement of the extinction coefficient at a wavelength of 510 nm is a prerequisite for the accurate analysis of the samples. The standard curve was plotted with the mass concentration of rutin on the *x*‐axis and the absorbancy on the *y*‐axis. The linear regression equation obtained was *y* = 5.9056*x* − 0.0025 (*R* ^2^ = 0.9994). A volume of 200 μL of the test solution is required for the measurement of the extinction coefficient, which is then used to calculate the total flavonoid content based on the standard curve.

(1) $$M=\left(C\times V\times n\right)÷m$$

where *M* is the total flavonoid content (mg/g DW); *C* is the concentration (mg/mL); *V* is the volume (mL); *n* is the dilution factor; and *m* is the mass (g).

#### 2.4.2 Determination of Total Phenolic Content

The method described by Madhava Naidu et al. (2011) was followed with minor modifications. Gallic acid was used as the reference standard, and the Folin–Ciocalteu colorimetric assay was used to determine TPC, expressed as gallic acid equivalents. The assay also responds to flavonoids and other reducing substances; TPC is therefore an operational colorimetric measure, and TFC and TPC are not independent compound pools. Accurately pipette 200 μL of gallic acid standard solutions of varying concentrations, add 0.5 mL of Folin–Ciocalteu reagent, mix thoroughly, and allow to react for 5 min. Then, add 1.5 mL of 20% Na_2_CO_3_ solution, make up to 10 mL with distilled water, and react in a 75°C water bath in the dark for 10 min. Measure the absorbance at a wavelength of 765 nm. Plot a standard curve with the mass concentration of gallic acid on the *x*‐axis and absorbance on the *y*‐axis, yielding the linear regression equation *y* = 49.229*x* + 0.0075 (*R* ^2^ = 0.9991). Take 200 μL of the test solution and measure the absorbance using the same method. Calculate the total phenolic content based on the standard curve, and perform the calculation according to Equation (2).

(2) $$M=\left(C\times V\times n\right)÷m$$

where *M* is the total phenolic content (mg/g DW); *C* is the concentration (mg/mL); *V* is the volume (mL); *n* is the dilution factor; and *m* is the mass (g).

### 2.5 Assessment of Antioxidant Activity In Vitro

#### 2.5.1 DPPH Radical Scavenging Assay

We used vitamin C as the positive control. A 0.1 mmol/L DPPH solution was prepared. We also used 1.5 mL of LLB extract at different concentrations (0.2, 0.4, 0.6, 0.8, and 1.0 mg/mL) to make a mixture with 0.75 mL of the DPPH solution. After waiting 30 min in the dark, the absorbance was measured at a wavelength of 517 nm and recorded as *A*_1_. Then, an equal amount of the extract was mixed with 0.75 mL of anhydrous ethanol. After waiting in the dark, the absorbance was measured and recorded as *A*_2_. Finally, 1.5 mL of anhydrous ethanol was used instead of the extract and mixed with the DPPH solution. The absorbance was measured and recorded as *A*_0_. The DPPH radical scavenging rate was calculated using Equation (3) (Wang et al. 2013). The stated extract concentrations refer to the extract solution before mixing with the assay reagent (1.5 mL extract +0.75 mL DPPH solution).

(3) $$\text{DPPH radical scavenging rate}\%=\left[1-\left(A_{1}-A_{2}\right)÷A_{0}\right]\times 100\%$$

#### 2.5.2 ABTS Radical Scavenging Assay

ABTS‐radical‐scavenging activity was determined according to Jing et al. (2015), with ascorbic acid used as the positive control. A 100 μL aliquot of LLB extract at concentrations of 0.2, 0.4, 0.6, 0.8, and 1.0 mg/mL was added to 3.9 mL of ABTS working solution. The mixture was incubated in the dark for 30 min, and absorbance was measured at 734 nm and recorded as *A*_2_. For the control, 100 μL of anhydrous ethanol was substituted for the extract, and the absorbance was recorded as *A*_1_. The stated extract concentrations refer to the extract solution before mixing with the assay reagent (0.1 mL extract + 3.9 mL ABTS working solution). ABTS‐radical‐scavenging activity was calculated using Equation (4):

(4) $$\text{ABTS radical scavenging rate}\%=\left[\left(A_{1}-A_{2}\right)÷A_{1}\right]\times 100\%$$

#### 2.5.3 Hydroxyl Radical Scavenging Assay

Take 0.3 mL of FeSO_4_ (7.5 mmol/L), 0.3 mL of H_2_O_2_ (7.5 mmol/L), 0.3 mL of salicylic acid (7.5 mmol/L, dissolved in anhydrous ethanol), and 1.0 mL of LLB extract at different concentrations (0.2, 0.4, 0.6, 0.8, and 1.0 mg/mL). Mix thoroughly and react in a 37°C water bath for 30 min; measure the absorbance at 510 nm and record as *A*_1_; replace the H_2_O_2_ with 0.3 mL distilled water and measure using the same method, recording the result as *A*_2_; replace the extract with 1.0 mL distilled water and measure using the same method, recording the result as *A*_0_ (Smirnoff and Cumbes 1989). Ascorbic acid was used as the positive control (5). The stated extract concentrations refer to the extract solution before mixing with the assay reagents (1.0 mL extract plus 0.9 mL of the other reaction components).

(5) $$\text{Hydroxyl radical scavenging rate}\%=\left[1-\left(A_{1}-A_{2}\right)÷A_{0}\right]\times 100\%$$

#### 2.5.4 Total Reducing Power Assay

The method described by Liu et al. (2021) was followed with minor modifications. Take 1 mL of a LLB extract solution of varying concentrations, add 0.2 mL of 0.2 mol/L phosphate buffer (pH 6.6) and 0.5 mL of 5% K_3_[Fe(CN)_6_], mix thoroughly, and react in a 50°C water bath for 20 min. Cool rapidly, add 1 mL of 10% trichloroacetic acid, and then centrifuge at 5000 r/min for 10 min. Take 1.5 mL of the supernatant, add 0.2 mL of 1% FeCl_3_ and 3 mL of distilled water, allow it to stand for 10 min, and measure the absorbance at 700 nm. Using VC as the positive control, a higher absorbance indicates stronger reducing capacity.

### 2.6 Optimization of UAE Parameters

#### 2.6.1 Single‐Factor Experiments

The UAE method was employed to extract TFC and TPC from LLB. The following factors were investigated: extraction temperature (15°C, 30°C, 45°C, 60°C, and 75°C), extraction time (15, 30, 45, 60, and 75 min), solid‐to‐liquid ratio (1:10, 1:25, 1:40, 1:55, and 1:70 g/mL), and ultrasonic power (300, 450, 600, 750, and 900 W). The baseline conditions for the single‐factor experiments were set as follows: extraction temperature 30°C, extraction time 15 min, solid‐to‐liquid ratio 1:25 g/mL, and ultrasonic power 300 W. When investigating a single factor, the other factors were kept constant. The absorbance of the resulting extracts was measured using the standard curve method, and the TFC and TPC contents were calculated.

The four variables were selected because they are principal controllable UAE parameters that may affect mass transfer and the stability of phenolic constituents. Ultrasound power affects cavitation intensity. Insufficient power may not adequately disrupt plant tissues, whereas excessive power can generate local heating and promote degradation of sensitive constituents (Ozdemir et al. 2026; Ponphaiboon et al. 2026). Extraction temperature affects solvent viscosity, solubility, and diffusion, although excessive heating may accelerate phenolic oxidation or degradation (Gheisary et al. 2025). Extraction time controls contact between the solvent and plant matrix. A short extraction time may be insufficient for solute release, whereas prolonged treatment may not further improve yield and may compromise compound stability (Ponphaiboon et al. 2026). The solid‐to‐liquid ratio determines the solvent concentration gradient and extraction capacity; an insufficient solvent volume can limit mass transfer, whereas excessive solvent increases reagent use and the downstream concentration burden.

The factor ranges for response surface optimization were selected from the single‐factor profiles to encompass the response maxima while avoiding inadequate extraction, excessive solvent use, and possible thermal or ultrasonic degradation. Because the single‐factor maxima differed between TFC and TPC, separate factor ranges were used for the two Box–Behnken designs (Table 1).

**TABLE 1:** Factors and levels in response surface test.

Factor		Measured indicators (mg/g DW)	−1	0	1
A	Time (min)	TFC	45	60	75
		TPC	30	45	60
B	S/S ratio (g/mL)	TFC	1:10	1:25	1:40
		TPC	1:10	1:25	1:40
C	Power (W)	TFC	450	600	750
		TPC	300	450	600
D	Temperature (°C)	TFC	45	60	75
		TPC	45	60	75

#### 2.6.2 Response Surface Optimization Experiments

Based on the Box–Behnken experimental design principle and the results of the single‐factor experiments, two independent four‐factor, three‐level response surface optimization experiments were conducted separately for TFC and TPC. For each experiment, the independent variables were ultrasonic extraction time (A), solid‐to‐liquid ratio (B), ultrasonic power (C), and extraction temperature (D), with the aim of determining the optimal extraction conditions for the respective response. The factor levels for the TFC and TPC experiments are presented separately in Table 1. Each Box–Behnken design comprised 29 experimental runs. Multiple linear regression analysis was used to estimate the regression coefficients, and the experimental data were fitted to a second‐order polynomial equation. The model equation is as follows:

(6) $$y=\beta_{0}+\sum_{i=1}^{4}\beta_{i}X_{i}+\sum_{i<j}^{4}\beta_{\mathrm{ij}}X_{i}X_{j}+\sum_{i=1}^{4}\beta_{\mathrm{ii}}X_{i}^{2}$$

*Y* is the response variable, *β*_0_ is the intercept (constant term), *βᵢ* is the coefficient of the linear term for the *i*‐th independent variable, *βᵢⱼ* is the coefficient of the interaction term between the *i*‐th and *j*‐th independent variables (where *i* < *j* to avoid repetition), and *βᵢᵢ* is the coefficient of the quadratic term for the *i*‐th independent variable. The model is evaluated using analysis of variance (ANOVA) and various goodness‐of‐fit measures to verify its adequacy in describing the relationship between the independent variables and the response variables (including TPC and TFC).

### 2.7 Statistical Analysis

Experimental data were tabulated using Microsoft Excel 2016, and graphs were prepared using Origin 2021. Statistical analyses were performed using SPSS version 24.0, and response surface modeling was conducted using Design‐Expert version 11. All experiments were performed in triplicate, and data are presented as mean ± standard deviation (SD). ANOVA was used to evaluate differences among treatments and to assess the significance of linear, interaction, and quadratic terms in the response surface models. When ANOVA indicated a significant overall effect, pairwise comparisons were performed using the least significant difference (LSD) test. Model adequacy was evaluated using the coefficient of determination (*R* ^2^), adjusted *R* ^2^, predicted *R* ^2^, coefficient of variation, lack‐of‐fit test, residual diagnostics, and predicted‐versus‐actual plots. Differences were considered statistically significant at *p* < 0.05 and highly significant at *p* < 0.01.

### 2.8 AI Use Statement

During the preparation of this manuscript, the authors used ChatGPT (OpenAI) to assist with language editing, Chinese‐to‐English translation, and improving the clarity and organization of selected text. Artificial intelligence was not used to generate, manipulate, or analyze experimental data, produce research results, or make scientific decisions. All AI‐assisted content was critically reviewed, edited, and verified by the authors. The authors take full responsibility for the accuracy, integrity, interpretation, and conclusions of the manuscript.

## 3 Results and Discussion

### 3.1 Comparison of Methods for Extracting Phenolic Compounds From LLB

The TFC and TPC contents of LLB extracts were evaluated using 70% ethanol under the tested operating conditions for RE, MAE, and UAE. As shown in Table 2, UAE at 600 W produced the highest TFC content, whereas UAE at 450 W produced the highest TPC content among the evaluated treatments. However, extraction temperatures were not matched across methods: RE was performed at 45°C–75°C, whereas MAE and UAE were performed at 30°C. Therefore, the observed differences reflect the combined effects of extraction method and method‐specific operating conditions rather than the effect of extraction method alone.

**TABLE 2:** TFC and TPC contents of LLB extracts obtained using different extraction methods under the tested operating conditions.

Extraction method	Extraction conditions	TFC (mg/g DW)	TPC (mg/g DW)
RE	45°C	28.73 ± 0.11^b^	18.38 ± 0.18^c^
	60°C	31.12 ± 0.52^c^	21.11 ± 0.59^d^
	75°C	30.92 ± 0.88^c^	20.77 ± 0.38^d^
MAE	400 W	26.55 ± 0.63^a^	16.32 ± 0.75^b^
	500 W	33.85 ± 0.43e^f^	16.94 ± 0.61^b^
	600 W	32.30 ± 0.33c^d^	10.88 ± 0.67^a^
UAE	450 W	33.55 ± 0.77d^e^	25.16 ± 0.79^f^
	600 W	37.79 ± 0.49^g^	23.39 ± 0.51^e^
	750 W	35.08 ± 0.64^f^	23.28 ± 0.34^e^

*Note:* Values are presented as mean ± SD (*n* = 3). Different lowercase letters within the same column indicate significant differences among treatments according to one‐way ANOVA followed by the LSD test (*p* < 0.05).

Extraction efficiency depends on both the solvent system and the extraction method. In conventional reflux extraction, the plant–solvent mixture is heated, and the solvent vapor is condensed and returned to the extraction vessel, thereby limiting solvent loss during heating (Bitwell et al. 2023; Shi et al. 2022). Li et al. (2025) used heat reflux extraction to recover polyphenols and flavonoids from hempseed threshing residues. Sheng et al. (2013) optimized the reflux extraction of total flavonoids from Flos Populi using a Box–Behnken design and response surface methodology. Microwave‐assisted extraction relies primarily on dielectric heating generated by ion migration and dipole rotation. Microwave energy can rapidly heat polar molecules in the solvent and plant matrix, which may disrupt cellular structures and enhance the release of target constituents (Nguyen et al. 2021). Compared with conventional solvent extraction, MAE may reduce extraction time and solvent consumption under appropriately controlled conditions (Sebastián et al. 2026). Terpinc et al. (2023) compared MAE and accelerated solvent extraction for recovering phenolic compounds from wild strawberry leaves.

Under the tested operating conditions, UAE showed the best overall extraction performance among the evaluated treatments. Ultrasound‐assisted extraction promotes mass transfer through acoustic cavitation, agitation, and disruption of plant tissues, thereby facilitating solvent penetration into the plant matrix and the release of target constituents. Compared with conventional extraction methods, UAE may increase extraction efficiency and reduce processing time while operating at relatively moderate bulk temperatures (Lai et al. 2010). Pollini et al. (2019) compared UAE, MAE, and maceration for phenolic recovery from *Lycium barbarum* leaves and reported that methanolic UAE was the most effective treatment among the tested conditions. Consistent with this observation, UAE produced the highest TFC and TPC contents among the evaluated treatments in the present study. The highest TFC content was obtained at an ultrasound power of 600 W, whereas the highest TPC content was obtained at 450 W. These different optima indicate that ultrasound power should be tailored to the target response. Excessively high ultrasound power or prolonged sonication may increase heating and promote cavitation‐related radical formation, potentially causing oxidation or degradation of sensitive bioactive constituents (Islam et al. 2023; Shen et al. 2023). Conversely, insufficient ultrasound power may weaken cavitation‐induced mechanical effects and cell‐wall disruption, thereby limiting the release and mass transfer of target compounds (Yusoff et al. 2022).

Previous studies of LLB have focused primarily on the isolation and characterization of individual chemical constituents and the evaluation of antioxidant activities of crude extracts. Under the optimized UAE conditions established in the present study, TFC and TPC reached 40.58 ± 0.94 mg/g DW and 26.38 ± 0.57 mg/g DW, respectively, indicating that the optimized process enabled effective recovery of phenolic bioactive constituents from LLB. For comparison, Wu et al. (2022) used response surface methodology to optimize UAE of total phenols from alfalfa ( *Medicago sativa* ) and reported a phenolic content of 15.76 mg/g DW using 50% acetone as the extraction solvent. Khan et al. (2025) reported a total polyphenol content of 44.82 mg gallic acid equivalents/g dry weight following UAE of green tea with 50% aqueous ethanol. The phenolic constituent contents obtained from LLB in the present study therefore fall within the range reported for UAE of other phenolic‐rich plant materials. Nevertheless, such cross‐study comparisons should be interpreted only as contextual references and not as an absolute ranking of extraction efficiency. Plant species and tissues, cell‐wall architecture, solvent composition, solid‐to‐liquid ratio, ultrasound parameters, analytical procedures, reporting basis, and calibration standards may all substantially affect the measured values. In particular, the operational definitions and calibration systems for “total phenols,” “total polyphenols,” “total phenolic acids,” and “total flavonoids” are not fully interchangeable across studies.

### 3.2 Optimisation of the UAE Process

#### 3.2.1 Single‐Factor Experiments

As shown in Figure 1, both TFC and TPC increased initially and subsequently declined as the level of each factor increased. At an ultrasound power of 450 W, TPC reached its maximum value of 25.16 mg/g DW (Figure 1A). In contrast, TFC reached its maximum value of 37.79 mg/g DW at 600 W. Both responses differed significantly from those obtained at the other power levels (*p* < 0.05). Appropriate ultrasound power can enhance extraction through acoustic cavitation. However, excessive power may increase the bulk solvent temperature and alter cavitation behavior, thereby reducing extraction efficiency (Ozdemir et al. 2026). Based on these results, power ranges of 300–600 W and 450–750 W were selected for the TPC and TFC response surface designs, respectively.

**FIGURE 1:**
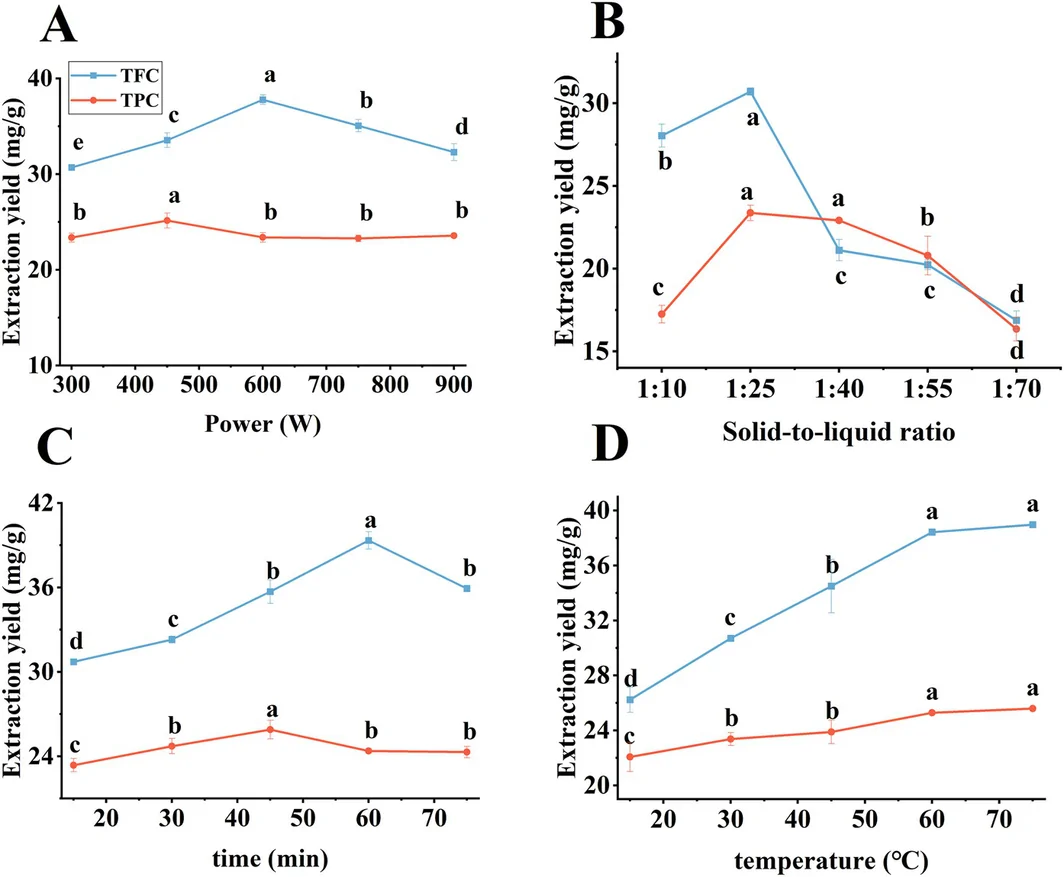
Results of single‐factor experiments for ultrasound‐assisted extraction. (A) Effect of ultrasound power; (B) effect of solid‐to‐liquid ratio; (C) effect of extraction time; (D) effect of extraction temperature. Different letters indicate significant differences (*p* < 0.05), while the same letters indicate no significant differences (*p* > 0.05). The same applies to the following figures.

As shown in Figure 1B, both TFC and TPC reached their maximum values at a solid‐to‐liquid ratio of 1:25 (g/mL), with contents of 30.71 and 23.37 mg/g DW, respectively. These values differed significantly from those obtained at the other tested ratios (*p* < 0.05). Therefore, a solid‐to‐liquid ratio range of 1:10–1:40 (g/mL) was selected for subsequent optimization of both responses. During extraction, phenolic constituents are transferred from the plant matrix to the solvent, primarily through concentration‐gradient‐driven mass transfer. An insufficient solvent volume may limit solvent penetration and reduce the mass‐transfer driving force (Tahir et al. 2025). In addition, the dense trichomes on LLB may retain solvent; therefore, an insufficient solvent volume can promote material aggregation and impair mixing. Conversely, an excessively high solvent volume increases solvent consumption and may co‐extract polar impurities, thereby increasing the burden of downstream purification (Chauhan et al. 2026). An appropriate solid‐to‐liquid ratio is therefore required to balance extraction efficiency and process economy.

As the ultrasonication time increased, the extraction yield rose significantly; this phenomenon is attributed to the cavitation effect of ultrasound, which promotes thorough interaction between the solvent and the sample. Nevertheless, it has been demonstrated that excessive ultrasonication time has the potential to compromise the molecular structure of the active components (Ponphaiboon et al. 2026). As demonstrated in Figure 1C, the TPC extraction yield attained its maximum value (25.9 mg/g DW) at an extraction time of 45 min; when the extraction time was 60 min, the TFC extraction yield reached its maximum value (39.34 mg/g DW), with a significant difference compared to other levels (*p* < 0.05). Consequently, the range of 30–60 min was selected for the investigation of TPC extraction, and the range of 45–75 min for the investigation of TFC extraction.

As demonstrated in Figure 1D, there was an increase in the extractable content of TFC and TPC with rising extraction temperature, before reaching a plateau at 60°C. The analysis revealed that at a temperature of 75°C, the extractable contents of total flavonoids and total phenolic content both attained their maximum values, with the former measuring 38.97 mg/g DW and the latter 25.6 mg/g DW, respectively. While no substantial disparities were observed in comparison to 60°C, notable variations were identified when evaluated against alternative temperature settings (*p* < 0.05). Appropriate temperatures have been shown to accelerate molecular diffusion, reduce mass transfer resistance, break down cell wall barriers, and increase solubility, thereby improving the extraction yield of active components (Gheisary et al. 2025). However, excessively high temperatures can damage the structure of active components, leading to a loss of activity. Phenolic compounds are susceptible to oxidation or polymerization reactions when exposed to elevated temperatures; hence, this study established a temperature range of 45°C–75°C for the extraction of TFC and TPC.

#### 3.2.2 Response Surface Optimisation Experiment

##### 3.2.2.1 Establishment of Response Surface Multiple Regression Equations

Based on the results of the single‐factor experiments and taking the extractable TFC content and TPC content as response variables, a response surface experimental design with four factors (extraction time, solid‐to‐liquid ratio, ultrasonic power, and extraction temperature) at three levels was employed to optimize the extraction process for the active components of LLB. The experimental design and results are shown in Tables 3 and 4. Through multiple regression analysis, quadratic polynomial regression equations were established with total flavonoid content (*Y*_1_) and total phenolic content (*Y*_2_) as response variables, as follows:

(7) $$\begin{array}{cc} Y_{1}=\; & 41.08+0.435A+0.6592B+0.1867C-0.3275D\\ & -0.7325\mathrm{AB}-0.1125\mathrm{AC}+0.92\mathrm{AD}-0.02\text{5BC}\\ & -0.29\mathrm{BD}-0.0425\mathrm{CD}-2.26A^{2}-2.03B^{2}\\ & -1.37C^{2}-1.56D^{2} \end{array}$$

(8) $$\begin{array}{cc} Y_{2}=\; & 26.64-0.8442A-0.1417B-0.2433C-0.8958D\\ & -0.32\text{5AB}+0.48\text{5AC}-1.03\mathrm{AD}+0.0025\mathrm{BC}\\ & +1.05\mathrm{BD}+0.1075\mathrm{CD}-1.05A^{2}-2.24B^{2}\\ & -1.59C^{2}-1.75D^{2} \end{array}$$

**TABLE 3:** Response surface optimisation scheme and results for the extraction conditions of TFC using ultrasound‐assisted extraction.

Experiment no.	A time (min)	B S/S ratio (g/mL)	C power (W)	D temperature (°C)	*Y*_1_ TFC (mg/g DW)
1	−1 (45 min)	−1 (1:10)	0 (600 W)	0 (60°C)	35.21
2	1 (75 min)	−1 (1:10)	0 (600 W)	0 (60°C)	37.13
3	−1 (45 min)	1 (1:40)	0 (600 W)	0 (60°C)	38.05
4	1 (75 min)	1 (1:40)	0 (600 W)	0 (60°C)	37.04
5	0 (60 min)	0 (1:25)	−1 (450 W)	−1 (45°C)	38.45
6	0 (60 min)	0 (1:25)	1 (750 W)	−1 (45°C)	38.51
7	0 (60 min)	0 (1:25)	−1 (450 W)	1 (75°C)	38.02
8	0 (60 min)	0 (1:25)	1 (750 W)	1 (75°C)	37.91
9	−1 (45 min)	0 (1:25)	0 (600 W)	−1 (45°C)	37.9
10	1 (75 min)	0 (1:25)	0 (600 W)	−1 (45°C)	37.24
11	−1 (45 min)	0 (1:25)	0 (600 W)	1 (75°C)	35.16
12	1 (75 min)	0 (1:25)	0 (600 W)	1 (75°C)	38.18
13	0 (60 min)	−1 (1:10)	−1 (450 W)	0 (60°C)	36.74
14	0 (60 min)	1 (1:40)	−1 (450 W)	0 (60°C)	38.03
15	0 (60 min)	−1 (1:10)	1 (750 W)	0 (60°C)	37.09
16	0 (60 min)	1 (1:40)	1 (750 W)	0 (60°C)	38.28
17	−1 (45 min)	0 (1:25)	−1 (450 W)	0 (60°C)	36.5
18	1 (75 min)	0 (1:25)	−1 (450 W)	0 (60°C)	37.7
19	−1 (45 min)	0 (1:25)	1 (750 W)	0 (60°C)	37.57
20	1 (75 min)	0 (1:25)	1 (750 W)	0 (60°C)	38.32
21	0 (60 min)	−1 (1:10)	0 (600 W)	−1 (45°C)	36.88
22	0 (60 min)	1 (1:40)	0 (600 W)	−1 (45°C)	38.8
23	0 (60 min)	−1 (1:10)	0 (600 W)	1 (75°C)	36.91
24	0 (60 min)	1 (1:40)	0 (600 W)	1 (75°C)	37.67
25	0 (60 min)	0 (1:25)	0 (600 W)	0 (60°C)	40.79
26	0 (60 min)	0 (1:25)	0 (600 W)	0 (60°C)	40.96
27	0 (60 min)	0 (1:25)	0 (600 W)	0 (60°C)	41.25
28	0 (60 min)	0 (1:25)	0 (600 W)	0 (60°C)	41.67
29	0 (60 min)	0 (1:25)	0 (600 W)	0 (60°C)	40.72

**TABLE 4:** Response surface optimisation scheme and results for the extraction conditions of TPC using ultrasound‐assisted extraction.

Experiment no.	A time (min)	B S/S ratio (g/mL)	C power (W)	D temperature (°C)	*Y*_2_ TPC (mg/g DW)
1	−1 (30 min)	−1 (1:10)	0 (450 W)	0 (60°C)	24.06
2	1 (60 min)	−1 (1:10)	0 (450 W)	0 (60°C)	23.52
3	−1 (30 min)	1 (1:40)	0 (450 W)	0 (60°C)	24.08
4	1 (60 min)	1 (1:40)	0 (450 W)	0 (60°C)	22.24
5	0 (45 min)	0 (1:25)	−1 (300 W)	−1 (45°C)	24.63
6	0 (45 min)	0 (1:25)	1 (600 W)	−1 (45°C)	23.61
7	0 (45 min)	0 (1:25)	−1 (300 W)	1 (75°C)	23.05
8	0 (45 min)	0 (1:25)	1 (600 W)	1 (75°C)	22.46
9	−1 (30 min)	0 (1:25)	0 (450 W)	−1 (45°C)	24.71
10	1 (60 min)	0 (1:25)	0 (450 W)	−1 (45°C)	24.61
11	−1 (30 min)	0 (1:25)	0 (450 W)	1 (75°C)	24.72
12	1 (60 min)	0 (1:25)	0 (450 W)	1 (75°C)	20.51
13	0 (45 min)	−1 (1:10)	−1 (300 W)	0 (60°C)	22.95
14	0 (45 min)	1 (1:40)	−1 (300 W)	0 (60°C)	22.97
15	0 (45 min)	−1 (1:10)	1 (600 W)	0 (60°C)	22.23
16	0 (45 min)	1 (1:40)	1 (600 W)	0 (60°C)	22.26
17	−1 (30 min)	0 (1:25)	−1 (300 W)	0 (60°C)	25.4
18	1 (60 min)	0 (1:25)	−1 (300 W)	0 (60°C)	22.71
19	−1 (30 min)	0 (1:25)	1 (600 W)	0 (60°C)	24.49
20	1 (60 min)	0 (1:25)	1 (600 W)	0 (60°C)	23.74
21	0 (45 min)	−1 (1:10)	0 (450 W)	−1 (45°C)	24.88
22	0 (45 min)	1 (1:40)	0 (450 W)	−1 (45°C)	22.54
23	0 (45 min)	−1 (1:10)	0 (450 W)	1 (75°C)	20.82
24	0 (45 min)	1 (1:40)	0 (450 W)	1 (75°C)	22.67
25	0 (45 min)	0 (1:25)	0 (450 W)	0 (60°C)	26.69
26	0 (45 min)	0 (1:25)	0 (450 W)	0 (60°C)	26.67
27	0 (45 min)	0 (1:25)	0 (450 W)	0 (60°C)	26.5
28	0 (45 min)	0 (1:25)	0 (450 W)	0 (60°C)	26.95
29	0 (45 min)	0 (1:25)	0 (450 W)	0 (60°C)	26.39

As shown in Table 5, the regression models for both TFC and TPC were highly significant (*p* < 0.01), while the lack‐of‐fit terms were not significant (*p* > 0.05). For the total flavonoids model, the correlation coefficient (*R* ^2^) was 0.9783, and the adjusted coefficient of determination (*R* ^2^_Adj_) was 0.9567, indicating that the model explained 95.67% of the response variability; the predicted coefficient (*R* ^2^_Pred_) was 0.9093, and the coefficient of variation (CV%) was 0.8921. For the total phenolic content model, *R* ^2^ was 0.9743, *R* ^2^_Adj_ was 0.9486, indicating that the model explained 94.86% of the response variability; *R* ^2^_Pred_ was 0.8612, and CV% was 1.63. All indices were within normal ranges. The points on the normal probability plot of residuals were distributed along the ideal fitting line, and the points on the predicted versus actual plot were also clustered around the ideal fitting line (Figure 2), indicating that the models exhibited high goodness‐of‐fit and reliability, and could be effectively used to analyze and predict the extraction conditions for LLB. For TFC, *A* (extraction time), *B* (solid‐to‐liquid ratio), *D* (extraction temperature), AB, AD, *A* ^2^, *B* ^2^, *C* ^2^, and *D* ^2^ showed highly significant effects (*p* < 0.01). For TPC content, *C* (ultrasound power) and AC had significant effects (*p* < 0.05); while *A*, *D*, AD, BD, *A* ^2^, *B* ^2^, *C* ^2^, and *D* ^2^ exhibited highly significant effects (*p* < 0.01). The order of influence on TFC was: solid‐to‐liquid ratio > extraction time > extraction temperature > ultrasound power; the order of influence on TPC content was: extraction temperature > extraction time > ultrasound power > solid‐to‐liquid ratio.

**TABLE 5:** Ultrasound‐assisted extraction of TFC and TPC by response surface regression model analysis of variance.

Source	Sum of squares		Df		Mean square		*F*‐value		*p*	
	*Y*_1_	*Y*_2_	*Y*_1_	*Y*_2_	*Y*_1_	*Y*_2_	*Y*_1_	*Y*_2_	*Y*_1_	*Y*_2_
Model	73.05	80.39	14	14	5.22	5.74	45.19	37.91	< 0.0001	< 0.0001
*A*‐time	2.27	8.55	1	1	2.27	8.55	19.66	56.45	0.0006	< 0.0001
*B*‐S/S ratio	5.21	0.2408	1	1	5.21	0.2408	45.15	1.59	< 0.0001	0.228
*C*‐power	0.4181	0.7105	1	1	0.4181	0.7105	3.62	4.69	0.0778	0.0481
*D*‐temperature	1.29	9.63	1	1	1.29	9.63	11.15	63.57	0.0049	< 0.0001
AB	2.15	0.4225	1	1	2.15	0.4225	18.59	2.79	0.0007	0.1171
AC	0.0506	0.9409	1	1	0.0506	0.9409	0.4384	6.21	0.5186	0.0258
AD	3.39	4.22	1	1	3.39	4.22	29.32	27.88	< 0.0001	0.0001
BC	0.0025	0	1	1	0.0025	0	0.0217	0.0002	0.8851	0.9899
BD	0.3364	4.39	1	1	0.3364	4.39	2.91	28.97	0.1099	< 0.0001
CD	0.0072	0.0462	1	1	0.0072	0.0462	0.0626	0.3052	0.8061	0.5894
*A* ^2^	33.11	7.13	1	1	33.11	7.13	286.77	47.1	< 0.0001	< 0.0001
*B* ^2^	26.75	32.69	1	1	26.75	32.69	231.64	215.82	< 0.0001	< 0.0001
*C* ^2^	12.16	16.3	1	1	12.16	16.3	105.34	107.58	< 0.0001	< 0.0001
*D* ^2^	15.7	19.78	1	1	15.7	19.78	135.95	130.58	< 0.0001	< 0.0001
Residual	1.62	2.12	14	14	0.1155	0.1515				
Lack of fit	1.01	1.94	10	10	0.1011	0.1939	0.6687	4.27	0.7246	0.0872
Pure error	0.6051	0.1816	4	4	0.1513	0.0454				
Cor total	74.66	82.51	28	28						
*R* ^2^	0.9783	0.9743								
*R* ^2^_Adj_	0.9567	0.9486								
*R* ^2^_Pred_	0.9093	0.8612								
CV%	0.8921	1.63								

**FIGURE 2:**
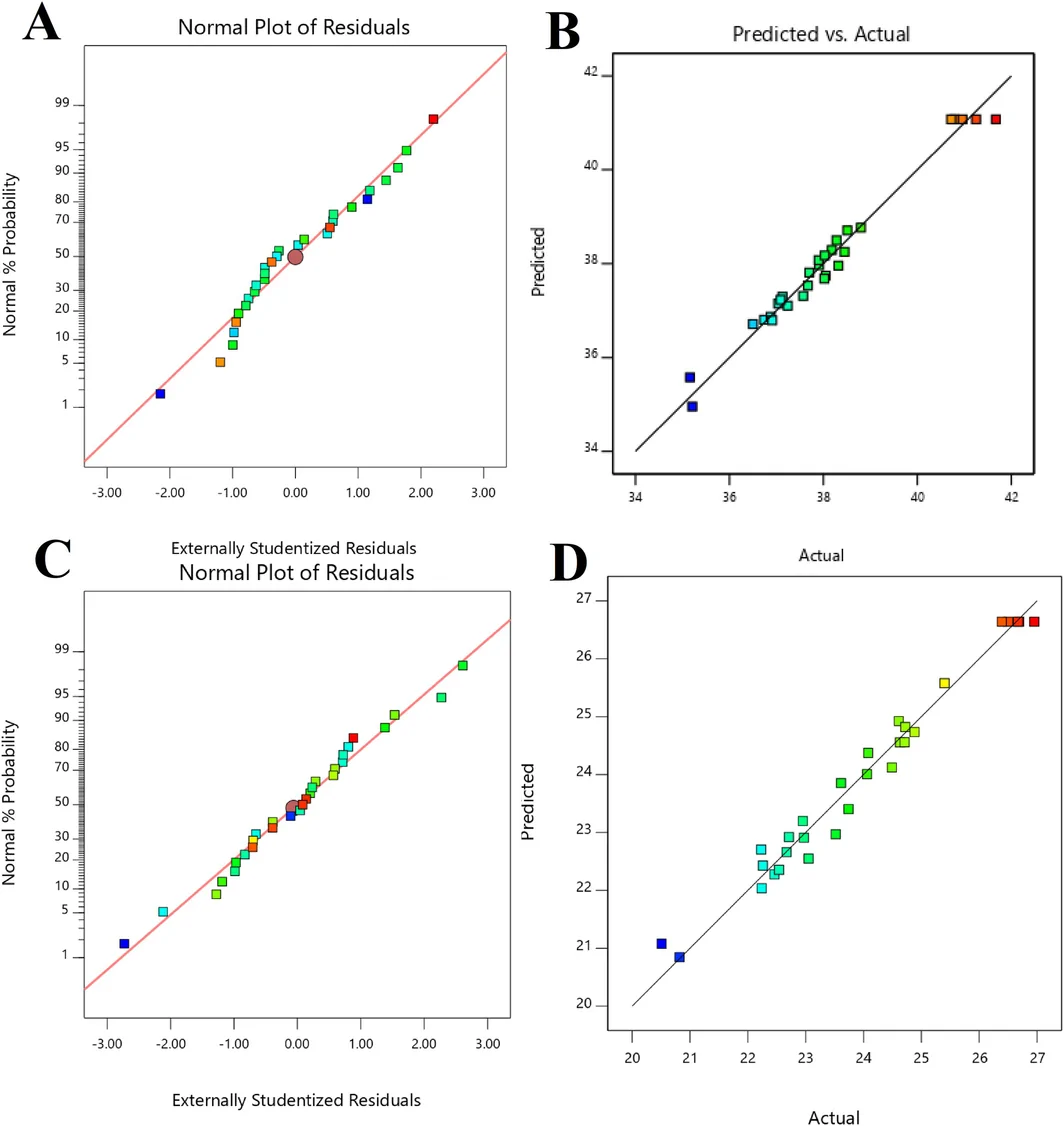
Normal probability plots of residuals and predicted versus actual values. (A) Normal probability plot of residuals for TFC; (B) Predicted versus actual values for TFC; (C) Normal probability plot of residuals for TPC; (D) Predicted versus actual values for TPC.

##### 3.2.2.2 Response Surface Interaction Analysis

As Figures 3 and 4 show, when one factor is held constant, the extractable content of TFC and TPC in LLB first increases and then decreases as the levels of the other factors increase. Combining the contour plots with the steepness of the three‐dimensional response surface plots reveals that the interactions between extraction time and both the solid‐to‐liquid ratio and the extraction temperature significantly influence the TFC extraction yield (*p* < 0.05), as shown in Figure 3. Figure 4 shows that the interactions between extraction time and ultrasonic power, extraction time and extraction temperature, and solid‐to‐liquid ratio and extraction temperature significantly affect the TPC extraction yield (*p* < 0.05). However, the interactions between the remaining factor combinations and the TFC and TPC extraction yields were not significant (*p* > 0.05).

**FIGURE 3:**
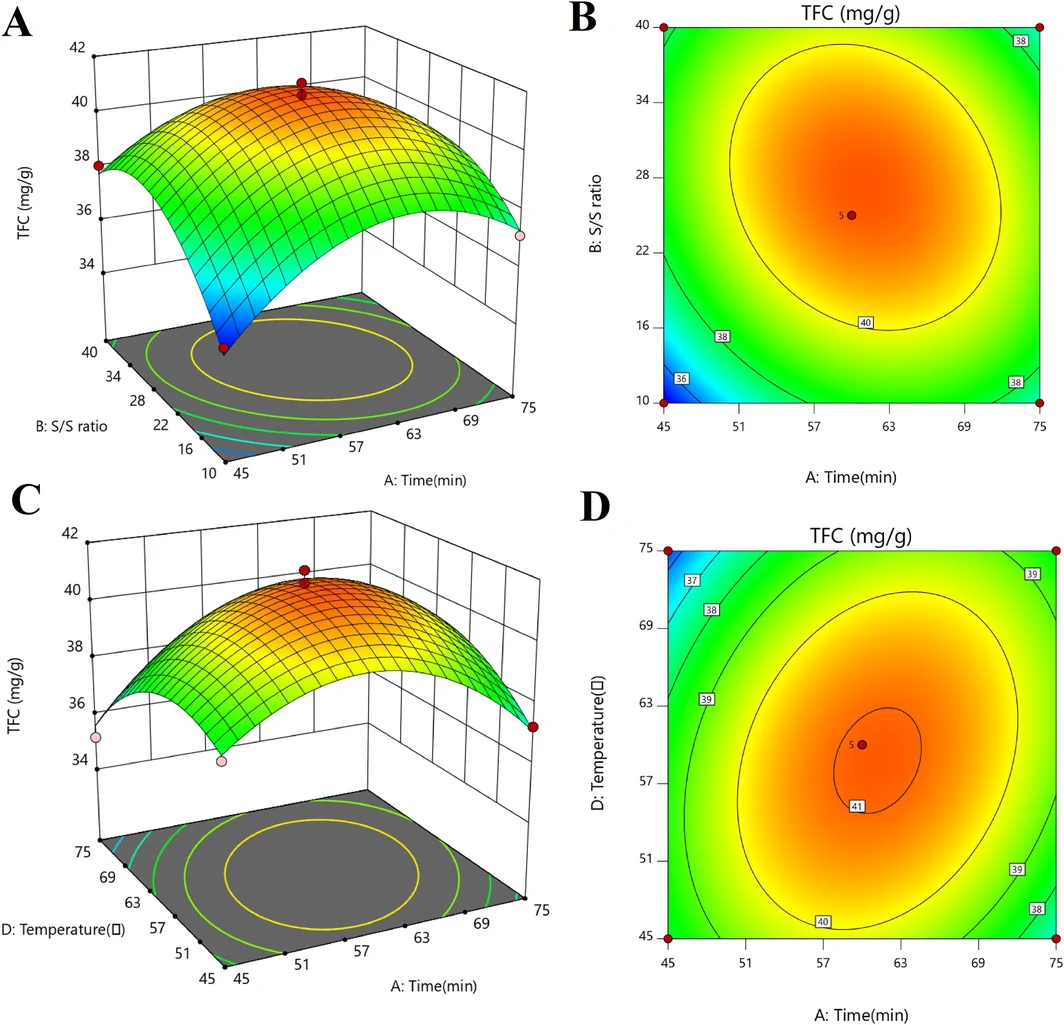
Effects of the interaction of various factors on the extraction content of TFC. (A) 3D response surface plot of extraction time and solid–liquid ratio; (B) Contour plot of extraction time and solid–liquid ratio; (C) 3D response surface plot of extraction time and temperature; (D) Contour plot of extraction time and temperature.

**FIGURE 4:**
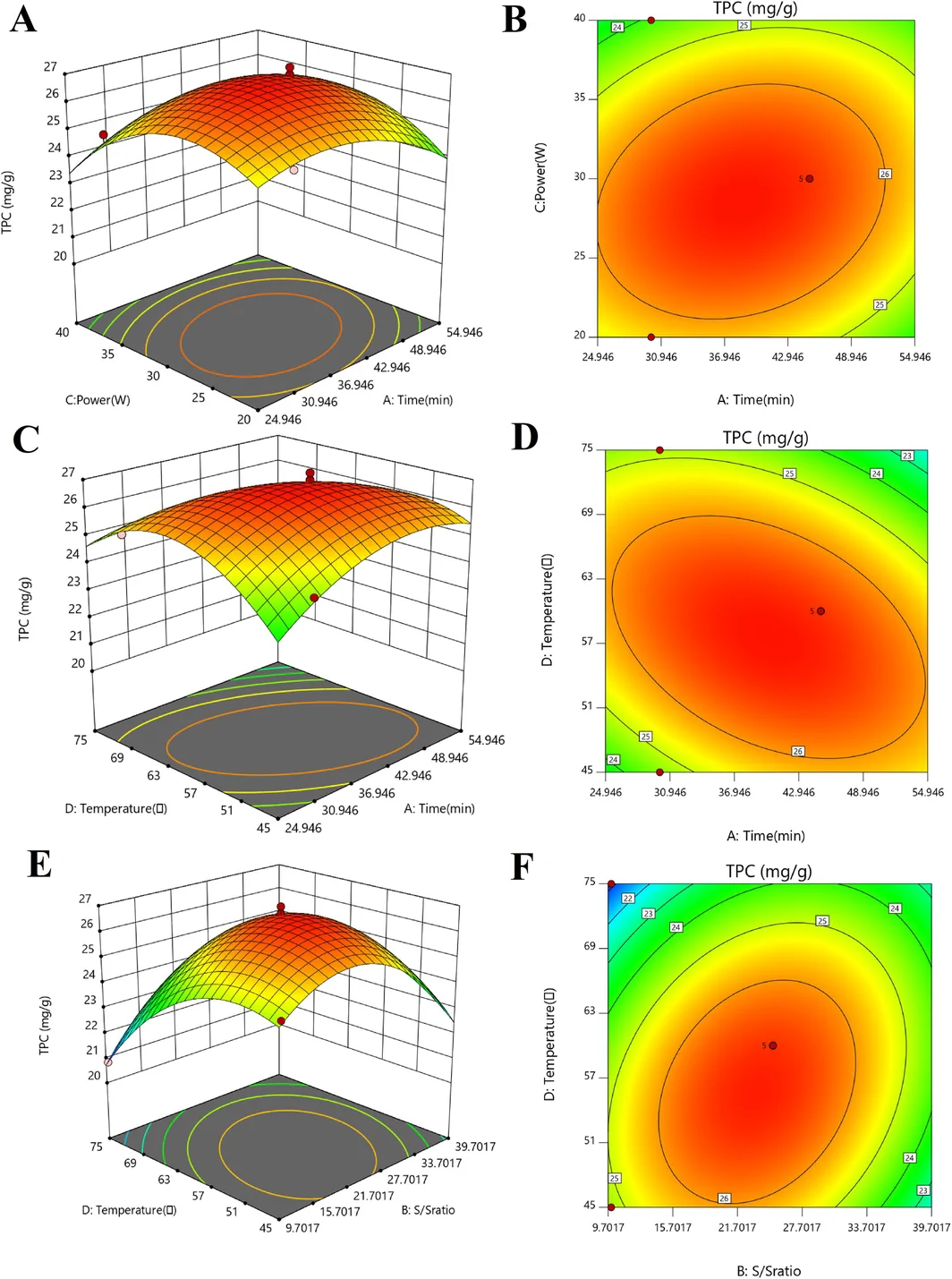
Effects of the interaction of various factors on the extraction content of TPC. (A) 3D response surface plot of extraction time and power; (B) Contour plot of extraction time and power; (C) 3D response surface plot of extraction time and temperature; (D) Contour plot of extraction time and temperature; (E) 3D response surface plot of solid–liquid ratio and temperature; (F) Contour plot of solid–liquid ratio and temperature.

In summary, the optimal extraction process for TFC was predicted based on an analysis of the model results and the response surface contour plots to be an extraction time of 60.71 min, a solid‐to‐liquid ratio of 1:27.417 g/mL, an ultrasonic power of 609.975 W, and an extraction temperature of 58.395°C, with a predicted extraction yield of 41.165 mg/g DW. To ensure practical feasibility, these parameters were adjusted as follows: Extraction time: 61 min; solid‐to‐liquid ratio: 1:27 g/mL; ultrasonic power: 610 W; and extraction temperature: 58°C. The optimal TPC extraction process was found to be an extraction time of 39.879 min, a solid‐to‐liquid ratio of 1:24.29 g/mL, an ultrasonic power of 429.735 W, and an extraction temperature of 57.377°C, with a predicted TPC extraction yield of 26.882 mg/g DW. The adjusted process parameters were an extraction time of 40 min, a solid‐to‐liquid ratio of 1:24 g/mL, an ultrasonic power of 430 W, and an extraction temperature of 57°C. Three parallel validation experiments were conducted under these optimal conditions, yielding measured TFC extraction yields of 40.58 ± 0.94 mg/g DW and TPC extraction yields of 26.38 ± 0.57 mg/g DW. These results were close to the predicted values, indicating that the process parameters obtained from model optimization are stable and reliable.

### 3.3 Application of the Optimal Extraction Method

#### 3.3.1 Effect of Different Grassland Types on the TFC and TPC Content of LLB

Using the optimal extraction process derived from single‐factor experiments and response surface optimisation, TFC and TPC were extracted from LLB collected from meadow grassland (MS) (Hulunbuir, Inner Mongolia), typical grassland (TS) (Chifeng, Inner Mongolia), and desert grassland (DS) (Ulanqab, Inner Mongolia) to investigate the influence of different grassland types on the content of bioactive compounds.

As shown in Table 6, the contents of active components in LLB varied significantly among different grassland types. In terms of TFC content, LLB from desert grassland exhibited the highest value at 46.15 mg/g DW, showing significant differences compared with other grassland types (*p* < 0.05); followed by typical grassland, with a content of 42.69 mg/g DW; while LLB from meadow grassland had the lowest TFC content at 38.78 mg/g DW. Regarding TPC content, LLB from meadow grassland had the highest value (23.77 mg/g DW), followed by typical grassland (23.18 mg/g DW), and desert grassland had the lowest (23.16 mg/g DW), with no significant differences observed among the three grassland types (*p* > 0.05). Comprehensive analysis indicates that desert grassland habitat is more favorable for the accumulation of TFC in LLB, whereas different ecological environments do not significantly affect the synthesis and accumulation of TPC.

**TABLE 6:** Contents of active ingredients in different grassland types of LLB.

Grassland type	Active ingredient content (mg/g DW)	
	TFC	TPC
MS	38.78 ± 0.49^c^	23.77 ± 0.87
TS	42.69 ± 0.85^b^	23.18 ± 0.22
DS	46.15 ± 0.77^a^	23.16 ± 0.21

*Note:* Different letters indicate significant differences (*p* < 0.05).

Relevant studies have also corroborated the regulatory role of ecological environments on plant active components. Xue et al. (2021) confirmed that *Artemisia annua* growing in desert grassland exhibited the highest accumulation of TFC, with decreasing levels observed in typical grassland and meadow grassland, respectively, which is highly consistent with the findings of the present study. Other scholars (Yan et al. 2004) have reported that Rhodiola species with superior medicinal quality are predominantly distributed in areas with soil available phosphorus deficiency, a pattern similar to the TFC accumulation observed in LLB in this study. A comparison was made of the differences in phenolic compound content between two *Bidens* species from different climates, highlighting that temperature, altitude, rainfall, and soil composition are key factors influencing phenolic content (Bimenyindavyi et al. 2023). In summary, multiple ecological factors—including altitude, temperature, precipitation, sunshine duration, accumulated temperature, soil fertility, grazing management, and frost‐free period—exert differential effects on the accumulation of active components in plants (Li et al. 2020).

#### 3.3.2 Antioxidant Activity Analysis of LLB From Different Grassland Types

In this study, the antioxidant activity of extracts from LLB collected from three grassland types—meadow grassland, typical grassland, and desert grassland—was evaluated. Ascorbic acid (VC) was used as a positive control. The analysis focused on determining the DPPH radical scavenging capacity, ABTS radical scavenging capacity, hydroxyl radical scavenging capacity, and total reducing power.

The DPPH radical scavenging activity of LLB extracts from different grassland types is shown in Figure 5A. A positive correlation was observed between the concentration of the extract and the DPPH radical scavenging rate, with the scavenging rate increasing steadily with higher concentrations. The antioxidant capacity, ranked in descending order, was as follows: DS > TS > MS. At an extract concentration of 0.8 mg/mL, the DPPH radical scavenging rates for all LLB exceeded 80%. Although the scavenging activity was slightly lower than that of the positive control, the LLB extracts still exhibited excellent DPPH radical scavenging capacity. The ABTS radical scavenging activity of extracts from different grassland types is shown in Figure 5B. The ABTS radical scavenging rate increased continuously with increasing extract concentration, indicating a positive correlation between concentration and ABTS radical scavenging activity. Among the three grassland types, DS exhibited the strongest scavenging capacity, followed by TS and MS. At an extract concentration of 1 mg/mL, the scavenging rates reached 26.45%, 15.37%, and 14.37%, respectively. As shown in Figure 5C, within the concentration range of 0.2–1 mg/mL, the hydroxyl radical scavenging rate increased continuously with increasing extract concentration, indicating a positive correlation between concentration and hydroxyl radical scavenging activity. Among the three grassland types, DS exhibited the strongest scavenging capacity, followed by MS and TS. At an extract concentration of 1 mg/mL, the scavenging rates reached 70.57%, 70.54%, and 54.6%, respectively. The reducing power of LLB extracts is shown in Figure 5D. Absorbance values increased with increasing extract concentration, indicating a positive correlation between extract concentration and total reducing power. Higher absorbance values reflect stronger reducing power. Although the total reducing power was lower than that of the positive control, the extracts still exhibited appreciable reducing capacity. The order of total reducing power was as follows: DS > MS > TS.

**FIGURE 5:**
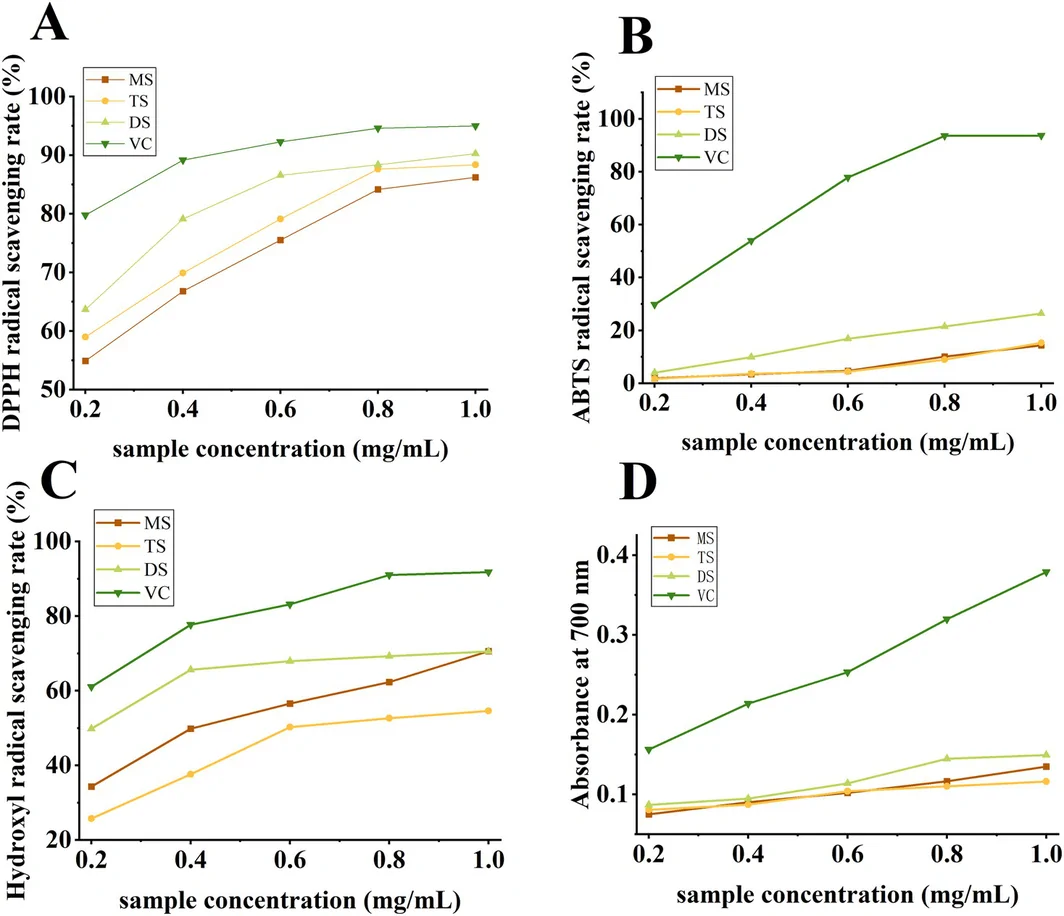
DPPH radical scavenging (A), ABTS radical scavenging (B), hydroxyl radical scavenging (C), and total reducing power (D) of LLB in different grass types.

The antioxidant capacity of plants is determined by their secondary metabolites, and differences in the structure, mechanism of action, and biological properties of these secondary metabolites contribute to variations in antioxidant activity. Flavonoids and phenolic acids, as secondary metabolites, possess the ability to scavenge free radicals in vivo and serve as natural antioxidants. In recent years, research on the antioxidant activity of LLB species has increased, demonstrating that LLB exhibits strong antioxidant properties. Wu et al. (2013) investigated the hydroxyl radical scavenging activity, total reducing power, and nitrite scavenging rate of different fractions of the ethanol extract of *Leontopodium* and found that the ethyl acetate fraction exhibited potent hydroxyl radical and nitrite scavenging capacity as well as strong reducing power. Zhao et al. (2021a, 2021b) evaluated the antioxidant activity of *Leontopodium* extracts with different polarities, and the results indicated that the DPPH and ABTS radical scavenging capacities varied among the extracts of different polarities. In this study, the antioxidant activity of LLB from different grassland types was evaluated, and the results revealed that different grassland types exhibited varying scavenging effects across the tested antioxidant indices. Another study reported that the total reducing power and DPPH radical scavenging capacity of *Auricularia auricula* differed among samples from different geographical origins (Su and Jian 2017). Various ecological environments can alter the morphological and structural characteristics of plants. Therefore, ecological factors such as temperature, altitude, and soil fertility may influence the antioxidant activity of plants. The strongest antioxidant activity observed in desert grassland may be attributed to the most severe abiotic stresses in this habitat, such as drought and intense radiation, which induce the accumulation of large quantities of secondary metabolites (e.g., flavonoids and phenolic acids) that serve as “antioxidant defense weapons” (Ilmiawati et al. 2025). LLB growing in meadow and typical grasslands had lower secondary metabolite levels than plants from the desert steppe. These differences may be associated with variation in water availability and plant–plant interactions. In resource‐limited environments, plants allocate more resources to defense, whereas in resource‐rich environments (e.g., meadow grasslands), plants tend to allocate more resources to growth (Jat et al. 2024; Javid et al. 2024; Şimşek et al. 2025).

## 4 Conclusion

In this study, LLB was used as the raw material to compare RE, MAE, and UAE for recovering TFC and TPC. Under the tested operating conditions, UAE showed the best overall extraction performance among the evaluated treatments and was therefore selected for response surface optimization. However, because extraction temperatures differed among RE (45°C–75°C), MAE (30°C), and UAE (30°C), the observed differences should be interpreted as combined effects of the extraction methods and their operating conditions rather than as the effect of extraction method alone. Response surface methodology was used to optimize UAE conditions separately for TFC and TPC. The optimal UAE conditions for TFC were an extraction time of 61 min, a solid‐to‐liquid ratio of 1:27 (g/mL), an ultrasound power of 610 W, and an extraction temperature of 58°C, yielding a TFC content of 40.58 ± 0.94 mg/g DW. The optimal conditions for TPC were an extraction time of 40 min, a solid‐to‐liquid ratio of 1:24 (g/mL), an ultrasound power of 430 W, and an extraction temperature of 57°C, yielding a TPC content of 26.38 ± 0.57 mg/g DW. Using the optimized UAE protocol, habitat‐associated variation in the phenolic constituents, and antioxidant activities of LLB was further evaluated. Samples collected from desert steppe (DS) exhibited the highest TFC content (46.15 mg/g DW), the strongest DPPH‐, ABTS‐, and hydroxyl‐radical‐scavenging activities, and the greatest reducing power. In contrast, TPC did not differ significantly among LLB samples collected from the three habitats (*p* > 0.05).

The optimized UAE protocol established in this study provides a laboratory‐scale basis for preparing LLB extracts containing phenolic constituents. Following further process validation, these extracts could be developed as standardized ingredients for functional foods and nutraceutical products, for which antioxidant capacity and compositional consistency are relevant quality attributes. The protocol may also inform the early‐stage development of LLB‐derived materials for pharmaceutical research. However, the present extraction results and in vitro antioxidant data alone do not support pharmaceutical application. Future studies should identify and quantify individual bioactive constituents, evaluate efficacy in relevant biological models, and establish toxicological safety, stability, dose–response relationships, and quality‐control criteria before pharmaceutical development is considered.

## Author Contributions

**Rule Yi:** conceptualization, methodology, writing – original draft, writing – review and editing, validation, formal analysis. **Junfeng Hao:** methodology, conceptualization, validation, writing – review and editing, project administration. **Jian Bao:** methodology, writing – review and editing. **Muqier Zhao:** methodology, writing – review and editing. **Zhijun Wang:** data curation, investigation. **Zhuna Bao:** formal analysis, investigation. **Gentu Ge:** conceptualization, supervision, funding acquisition, writing – review and editing, methodology.

## Funding

The present study was supported by a project on the Quality and Safety Regulation of Forage Products and the Development of Functional Products (YLXKZX‐NND‐004) and Scientific Research Start‐up Program for High Level, and Outstanding Doctoral Talents (NDYB2024‐14).

## Conflicts of Interest

The authors declare no conflicts of interest.

## Data Availability

The datasets generated and analyzed during the current study, including the raw extraction‐yield data and antioxidant‐assay data, are available from the corresponding author upon reasonable request. No supplementary data files are associated with this article.
